# Age-Related Differences in Bitter Taste and Efficacy of Bitter Blockers

**DOI:** 10.1371/journal.pone.0103107

**Published:** 2014-07-22

**Authors:** Julie A. Mennella, Danielle R. Reed, Kristi M. Roberts, Phoebe S. Mathew, Corrine J. Mansfield

**Affiliations:** Monell Chemical Senses Center, Philadelphia, Pennsylvania, United States of America; German Institute of Human Nutrition Potsdam-Rehbruecke, Germany

## Abstract

**Background:**

Bitter taste is the primary culprit for rejection of pediatric liquid medications. We probed the underlying biology of bitter sensing and the efficacy of two known bitter blockers in children and adults.

**Methods:**

A racially diverse group of 154 children (3-10 years old) and their mothers (N = 118) evaluated the effectiveness of two bitter blockers, sodium gluconate (NaG) and monosodium glutamate (MSG), for five food-grade bitter compounds (quinine, denatonium benzoate, caffeine, propylthiouracil (PROP), urea) using a forced-choice method of paired comparisons. The trial was registered at clinicaltrials.gov (NCT01407939).

**Results:**

The blockers reduced bitterness in 7 of 10 bitter-blocker combinations for adults but only 3 of 10 for children, suggesting that efficacy depends on age and is also specific to each bitter-blocker combination. Only the bitterness of urea was reduced by both blockers in both age groups, whereas the bitterness of PROP was not reduced by either blocker in either age group regardless of *TAS2R38* genotype. Children liked the salty taste of the blocker NaG more than did adults, but both groups liked the savory taste of MSG equally.

**Conclusions and Relevance:**

Bitter blocking was less effective in children, and the efficacy of blocking was both age and compound specific. This knowledge will pave the way for evidence-based strategies to help develop better-tasting medicines and highlights the conclusion that adult panelists and genotyping alone may not always be appropriate in evaluating the taste of a drug geared for children.

## Introduction

Unlike most adults, children have problems swallowing medicines in formulations such as pills or tablets, which have the advantage of encapsulating the active pharmaceutical ingredients (API). Instead, parents prefer that their children take medicine in liquid formulations, but here the central challenge becomes a “matter of taste” because APIs, by their nature, often are rejected because of their unpleasant tastes, with bitter being the primary culprit [1]. Most drugs work by changing physiological processes within cells, so APIs have the potential to be toxic when ingested in sufficient quantity. Bitter taste is thought to have evolved as a deterrent against ingestion of potentially harmful substances [2], which may explain why many drugs taste bitter. However, this bitter taste is also a major challenge to achieving medication compliance in pediatric patients.

Bitter perception starts at the level of the receptor. These are about 25 different bitter receptors (T2Rs) with genes clustered primarily on chromosomes 7 and 12 [3], [4]. The large number of receptors is needed because, of all the basic tastes, bitter is the most diverse [5]. Most T2Rs studied have binding profiles that involve several different bitter-tasting ligands [5], [6]. Likewise, a given bitter-tasting ligand can activate more than one T2R. This system accommodates the range of molecules that are perceived as bitter. Bitter taste is also perceptually diverse, and people differ markedly in their perceptions of the same compound. As an example, the perception of 6-*n*-propylthiouracil (PROP), which is primarily recognized by the most intensively studied T2R gene—the *TAS2R38* bitter receptor [7]—differs among people due to genetic variants within its receptor.

Bitter taste biology has opened new avenues to better understand taste perception in children and how unpleasant tastes might be reduced [8], [9]. Although several molecules that inhibit bitterness have been identified [10], [11], there are few peer-reviewed studies on the effectiveness of these bitter blockers (e.g., sodium salts, monosodium glutamate) in adults [8], [12], [13], [14], [15], [16], [17], and even fewer involving children [8]. To this end, the present study tested the efficacy of two food-grade bitter blockers, sodium gluconate (NaG) and monosodium glutamate (MSG), against five generally recognized as safe (GRAS) bitter agents in solution, in both children and adults. We used the same methodology in both age groups to determine if there were age-related differences in the efficacy of the blockers against quinine, denatonium benzoate (DB), caffeine, PROP, and urea, which represent a range of presumed classes of bitterness [18] but whose intensity can be as great as many oral formulations of medications, thus providing a useful model system for evaluating bitter blockers in children. Genetic variants of *TAS2R38* affect an individual's perception of the bitterness of PROP, even in children [7], [19], so each participant was genotyped for its receptor and phenotyped for PROP detection thresholds using a method that has shown reliability for children, adolescents, and adults [20]. Surprisingly, there have been no studies to date investigating whether the bitter taste of PROP can be blocked by either of these sodium salts.

## Methods

### Participants

Women with one or more children between the ages of 3 and 10 years were recruited from advertisements in local newspapers and Internet sites and from a database of past participants who asked to be notified of future research studies. During the telephone interview, the mothers were given detailed descriptions of the procedures for the “taste study” but were not told the goals of the study or hypotheses being tested. Women who were diabetic, pregnant, or lactating were not eligible; pregnancy tests were conducted on the day of testing to confirm they were not pregnant. The Office of Regulatory Affairs at the University of Pennsylvania approved all procedures. Written informed consent was obtained from each adult, and assent was obtained from each child 7 years of age or older. Mothers completed questionnaires regarding demographics and racial/ethnicity identity. The trial was registered at clinicaltrials.gov (NCT01407939).

### Test Stimuli

We selected for study five generally recognized as safe (GRAS) food-grade bitter compounds (Spectrum, New Brunswick, NJ) because of their prior use in research studies on adult psychophysical research [18]: 0.5 M urea, 0.008 M caffeine, 1.19×10^−4^ M quinine, 5.60×10^−4^ M propylthiouracil, and 4.92×10^−7^ M denatonium benzoate. We also selected two putative blockers based on previous studies of their effectiveness for some but not all bitter ligands [8], [12], [13], [14], [15], [16], [17]: 0.3 M sodium gluconate (NaG) and 0.1 M monosodium L-glutamate (MSG). NaG was chosen because previous research revealed that it was highly effective at suppressing the bitterness of urea and caffeine in adults and tasted less salty than sodium chloride [16]. The water used to prepare the solutions and as a rinsing solution was deionized Millipore-filtered water (Millipore Milli-Q Academic model). Solutions were stored in amber glass bottles and replaced at least every two weeks.

### Test Procedures

Following abstinence from eating for at least 1 hour, participants were tested individually in rooms specifically designed for sensory testing. As described below, after training, children and adults participated in the forced-choice, paired-comparison test to evaluate the bitterness of five food-grade (GRAS) bitter stimuli, separately and combined with each of the two blockers. Methods were identical for children and adults.

#### Training

To ensure that subjects understood the taste of bitterness (or bad/unpleasant taste), they were presented with three reference solutions that were identified as salty (0.3 M NaG, which is less salty than 0.3 M NaCl), sweet (0.3 M sucrose), and bitter (0.5 M urea). Then subjects were given a training session in which they received pairs of samples that differed in their sweetness and bitterness [8]; in some cases the more bitter solution was also sweet. Thus, subjects learned that in some cases the more bitter solution may be a complex mixture.

#### Psychophysical Testing

An age-appropriate, game-like task that minimized the impact of language development was used for both adults and children [8]. Using a forced-choice procedure, each subject was presented, in randomized order, six pairs of solutions for each bitter/blocker combination (see **Table S1**)**,** one pair at a time, and was asked to indicate which of the pair tasted more bitter. Included in the pairings was the comparison of the blocker alone and water to assess its contribution to the taste of bitter+blocker mixture. Procedures were identical for the children and their mothers, and testing for the five bitters was assessed with one blocker on one testing day and with the other on a separate testing day(s). Adults were presented with all pairs for a given blocker during one test session; children often required two sessions to complete a bitter blocker set.

Aliquots of 5 ml of each solution were presented in 30-ml polyethylene medicine cups. The order of presentation of the solutions was randomized within and between each pair of samples and between subjects. Subjects rinsed and expectorated with water two times after tasting each sample and four times between each pair. A 60-s interval separated each pair of solutions. During these intervals, subjects were offered a small unsalted cracker to cleanse their palate and a cup containing water for rinsing. To assess liking rather than bitterness, after all pairs had been presented, subjects were then presented with the putative blocker versus deionized water and asked which solution they preferred.

### PROP Taste Thresholds

We measured the lowest concentration of PROP at which subjects could recognize bitterness (bitterness threshold) for two reasons. First, we wanted to confirm the age-related differences in *TAS2R38* genotype–PROP phenotype relationship [20], [21], [22]. Second, if either of the blockers was effective in reducing PROP bitterness, we would then determine whether efficacy was related to genotype. To this end, we used a forced-choice ascending categorization procedure, developed by Anliker and colleagues [23] and modified by Mennella and colleagues [20], to accommodate the fact that children have difficulty with intensity/scaling measures. This method has proved to be reliable for children, adolescents, and adults: most children retested 9 months later demonstrate similar thresholds [20]. In brief, each subject was given four cups, one at a time, containing 5 ml of solution, which they were asked to taste and spit out after 5 s. The first cup was water, and the remaining cups were three different concentrations of PROP (56, 180, and 560 µM) presented in ascending order. To incorporate the task into a child-friendly game, subjects were told that if the solution tasted like water, they should give the cup to a stuffed toy Big Bird (a likeable puppet), but if it tasted “yucky” or bitter, they should give it to Oscar the Grouch (a grumpy puppet), so that he can “throw it in his trash can” [20] (both puppets are well-known television characters). Based on their responses, subjects were classified into four groups representing the lowest concentration, if any, given to Oscar the Grouch.

### Taste Receptor Genotyping

Children and their mothers provided DNA samples extracted from saliva following recommendations of the kit manufacturer (Genotek, Kanata, Canada). Subjects were typed for a genetic variant known to affect PROP perception (*TAS2R38*; *rs713598*, A49P) [20] using dye-labeled primers and probes purchased from Life Technologies (Grand Island, NY) and assayed using the StepOnePlus instrument from Applied Biosystems (Foster City, CA). The genotypes were checked for mother-child concordance, and allele frequencies were compared against earlier studies from a similar population [24].

### Statistical Analyses

For the paired-comparison data, we tested the null hypothesis that there were no systematic differences in children's or mothers' bitterness ranking between the four different solutions (i.e., bitter, bitter combined with blocker, blocker, and diluent). We analyzed the paired-comparison data for each blocker and each bitter stimulus separately. First, we determined the number of times (out of six possible pairings) that each subject chose each of the four solutions as tasting more bitter. From these data, we ranked the four solutions according to the subject's preferences, where 1 was the least chosen (least bitter) and 4 was the most chosen (most bitter). An example of the ranking procedure is shown in **Table S1**. Data obtained from mothers were analyzed separately from children. Separate Friedman Two-Way Nonparametric analyses were conducted on these bitter rankings derived from the paired-comparison data, one for each of the five bitter stimuli and for each of the two blockers. When significant, post hoc tests were performed to determine which differences among the solutions were significant [25].

The associations between the taste thresholds and *TAS2R38* genotypes were analyzed with chi-square analyses [25]; Yates' chi square was used when fewer than five subjects had a particular genotype. Follow-up partition analyses were then used to further examine these significant effects. All summary statistics are expressed as means ± SEM; the criterion for statistical significance for the omnibus and post-hoc tests was p<0.05.

## Results

### Subject Characteristics and Completion of the Tasks

**Table 1** provides the demographic characteristics of the sample, which reflect the diversity of race/ethnicity, family income, and adult educational levels of the city of Philadelphia [26]. The mothers were, on average, 33.7 (±0.7) years of age, and their children (87 girls, 67 boys) ranged in age from 3 to 10 years (7.7 ± 0.1), with 87 singletons, 27 sibling pairs, 3 sibling triads, and 1 sibling tetrad.

**Table 1:** Subject characteristics and completion of psychophysical tasks by age group.

Measure	Adults	Children
Sex	118 women	87 girls, 67 boys
Age, years (mean ± SEM)	33.7 ± 0.7	7.7 ± 0.1
Race/ethnicity [% (*n*)]		
White	19.5% (23)	13.6% (21)
Black	61.9% (73)	57.8% (89)
Hispanic/Latino/a	5.1% (6)	11.0% (17)
Asian	0.8% (1)	0.7% (1)
Other/more than one race	12.7% (15)	16.9% (26)
Socioeconomic data (adults only)		
Highest education level achieved: college graduate or higher [% (*n*/total answered)]	45.8% (54/118)	____
Completed/understood psychophysical testing [% (*n*/total tested)]^1^		
Paired-comparison task		
NaG	100% (116/116)	75.0% (114/152)
MSG	100% (110/110)	73.4% (105/143)
PROP threshold	98.3% (116/118)	94.8% (146/154)

Abbreviations: MSG, monosodium glutamate; NaG, sodium gluconate; PROP, propylthiouracil; SEM, standard error of the mean. ^1^Not all 118 mothers and 154 children participated in both sets of blocker paired comparisons.

Not all subjects participated in or completed all tasks. Of the 118 women tested, 108 participated in paired-comparison for both blockers. The remaining adults participated in the paired comparison task for just one blocker (NaG, n = 8; MSG, n = 2). Of the 154 children who participated, 152 were tested in the paired-comparison task for the blocker NaG and 143 for the blocker MSG. Of these 154 children, 22% children did not understand the task or comply with study procedures and thus were excluded from final analyses. Those excluded were younger than the remaining children (6.0 ± 0.3 vs. 8.2 ± 0.1 years; F(1,152) = 55.63; p<0.0001). All but eight of the children and two of the mothers completed the PROP threshold procedures. The DNA from three children and one mother was refractory to genotyping; missing data could be due to copy number or other variation in the *TAS2R38* genomic region or small variants in the sequences complementary to the primer or probe.

### Efficacy of Blockers by Bitter Agent and Age Group

For each of the five bitter agents, children and mothers ranked the bitter agent alone as tasting more bitter than water or either of the blockers alone (NaG or MSG; **Table 2**). When we analyzed the ranking data, we found that the blockers were effective in reducing the bitterness of some but not all bitters, and in some cases findings differed between children and adults. As shown in **Table 2** and summarized in **Table 3**, urea bitterness was reduced by both blockers, whereas the bitterness of PROP was not reduced by either blocker in any age or genotype group (**Table 4** **).** Caffeine and denatonium bitterness were reduced by one or both blockers, respectively, but for adults only. The bitterness of quinine was reduced by both blockers in adults but only by NaG in children. When asked which tasted better, the blocker or water, children were more likely to prefer the taste of NaG than were mothers (46.5% vs. 34.5%; p = 0.04), consistent with previous research^10^, but there was no age-related difference in preference for the taste of MSG (42.9 vs. 48.2%, p = 0.26).

**Table 2:** Efficacy of two putative bitter blockers: bitter rankings from forced-choice paired-comparison tests (mean ± SEM) and summary of post hoc analyses^1^.

Blocker = NaG	Bitter		Bitter+NaG	NaG	Diluent
Children	Urea	3.3±0.1^a^	2.5±0.1^b^	2.6±0.1^b^	1.6±0.1^c^
	Caffeine	3.3±0.1^a^	2.9±0.1^a^	2.1±0.1^b^	1.6±0.1^c^
	Quinine	3.5±0.1^a^	2.8±0.1^b^	2.0±0.1^c^	1.7±0.1^c^
	PROP	3.3±0.1^a^	2.9±0.1^a^	2.1±0.1^b^	1.7±0.1^b^
	Denatonium	3.5±0.1^a^	3.1±0.1^a^	1.8±0.1^b^	1.6±0.1^b^
Adults	Urea	3.6±0.1^a^	2.6±0.1^b^	2.3±0.1^b^	1.5±0.1^c^
	Caffeine	3.6±0.1^a^	3.1±0.1^b^	2.0±0.1^c^	1.4±0.04^d^
	Quinine	3.7±0.1^a^	2.8±0.1^b^	2.0±0.1^c^	1.5±0.1^c^
	PROP	3.3±0.1^a^	3.2±0.1^a^	2.0±0.1^b^	1.5±0.1^c^
	Denatonium	3.8±0.04^a^	3.1±0.04^b^	1.8±0.1^c^	1.3±0.05^d^
Blocker = MSG	Bitter		Bitter+MSG	MSG	Diluent
Children	Urea	3.3±0.1^a^	2.6±0.1^b^	2.4±0.1^b^	1.7±0.1^c^
	Caffeine	3.2±0.1^a^	3.1±0.1^a^	2.1±0.1^b^	1.6±0.1^b^
	Quinine	3.3±0.1^a^	2.9±0.1^a^	2.0±0.1^b^	1.8±0.1^b^
	PROP	3.0±0.1^a^	3.0±0.1^a^	2.3±0.1^b^	1.7±0.1^c^
	Denatonium	3.4±0.1^a^	3.1±0.1^a^	2.0±0.1^b^	1.5±0.1^b^
Adults	Urea	3.5±0.1^a^	2.5±0.1^b^	2.4±0.1^b^	1.5±0.1^c^
	Caffeine	3.5±0.1^a^	3.2±0.1^a^	2.0±0.1^b^	1.4±0.1^c^
	Quinine	3.7±0.05^a^	2.8±0.1^b^	2.0±0.1^c^	1.5±0.1^d^
	PROP	3.2±0.1^a^	3.1±0.1^a^	2.2±0.1^b^	1.5±0.1^c^
	Denatonium	3.7±0.05^a^	3.2±0.1^b^	1.8±0.05^c^	1.3±0.1^c^

1 All Friedman statistical tests of rank tests were significant at p≤0.0001. Abbreviations: SEM, standard error of the mean. Bitter compounds—0.5 M urea; 0.008 M caffeine; 1.19×10^−4^ M quinine; 5.60×10^−4^ M PROP (propylthiouracil); 4.92×10^−7^ M denatonium benzoate. Putative bitter blockers—NaG, 0.3 M sodium gluconate; MSG, 0.1 M glutamate. Diluent was distilled water. Blockers were compared separately against each bitter alone, bitter + blocker, and diluent. Rankings for bitterness range from 1 (least) to 4 (most); see Table S1 for ranking procedure. Different letters indicate rankings that are statistically different from each other within each row using p<0.05 as the statistical threshold for the omnibus and post-hoc tests.

**Table 3:** Summary of efficacy of NaG and MSG in reducing bitterness in adult and pediatric populations: analysis of the ranking data.

Bitter Agent	Blocker by Age Group			
	NaG		MSG	
	Children	Adults	Children	Adults
Urea	↓	↓	↓	↓
Caffeine	↔	↓	↔	↔
Quinine	↓	↓	↔	↓
Denatonium	↔	↓	↔	↓
PROP	↔	↔	↔	↔

Abbreviations: MSG, monosodium glutamate; NaGlu, sodium gluconate; PROP, propylthiouracil. ↓ signifies the blocker significantly reduced bitterness of a particular bitter agent as determined by Friedman statistical tests of ranks. ↔ signifies the blocker did not significantly reduce bitterness.

**Table 4:** Efficacy of blockers: summary of post hoc analyses* from bitter rankings in the paired-comparison test ^1^ by *TAS2R38* genotype for each of the two blockers when combined with PROP in each of the two groups: children and adults (means ± SEM).

Blocker = NaG	Genotype	PROP	PROP+NaG	NaG	Diluent
Children	AA	2.7±0.2^a^	2.4±0.2^a^	2.8±0.2^a^	2.1±0.2^a^
	AP	3.5±0.1^a^	3.1±0.1^a^	1.9±0.1^b^	1.6±0.1^b^
	PP	3.5±0.1^a^	3.2±0.1^a^	1.6±0.1^b^	1.7±0.1^b^
	All genotypes	3.3±0.1^a^	2.9±0.1^a^	2.1±0.1^b^	1.7±0.1^b^
Adults	AA	2.5±0.2^a,b^	3.0±0.1^a^	2.5±0.2^a,b^	1.9±0.2^b^
	AP	3.5±0.1^a^	3.3±0.1^a^	1.8±0.1^b^	1.4±0.1^b^
	PP	3.5±0.1^a^	3.4±0.1^a^	1.9±0.1^b^	1.2±0.1^b^
	All genotypes	3.3±0.1^a^	3.2±0.1^a^	2.0±0.1^b^	1.5±0.1^c^
Blocker = MSG	Genotype	PROP	PROP+MSG	MSG	Diluent
Children	AA	2.4±0.2^a^	2.8±0.2^a^	2.8±0.2^a^	2.0±0.2^a^
	AP	3.3±0.1^a^	3.0±0.1^a^	2.2±0.2^b^	1.5±0.1^c^
	PP	3.2±0.2^a^	3.1±0.2^a^	1.8±0.2^b^	1.9±0.2^b^
	All genotypes	3.0±0.1^a^	3.0±0.1^a^	2.3±0.1^b^	1.7±0.1^c^
Adults	AA	2.6±0.2^a^	2.7±0.2^a^	2.8±0.2^a^	1.9±0.2^a^
	AP	3.4±0.1^a^	3.2±0.1^a^	2.0±0.1^b^	1.4±0.1^c^
	PP	3.5±0.1^a^	3.3±0.1^a^	2.0±0.1^b^	1.2±0.1^b^
	All genotypes	3.2±0.1^a^	3.1±0.1^a^	2.2±0.1^b^	1.5±0.1^c^

Abbreviations: PROP, propylthiouracil; SEM, standard error of the mean. Putative bitter blockers—NaG, 0.3 M sodium gluconate; MSG, 0.1 M glutamate. Rankings for bitterness range from 1 (least) to 4 (most); see Table S1 for ranking procedure. Different letters indicate rankings that are statistically different from each other within each row. Genotypes (AA, AP, PP) refer to the bitter-sensitive (P) and bitter-insensitive (A) forms of A49P alleles of the *TAS2R38* bitter receptor gene.^1^All Friedman statistical tests of rank tests were significant at p≤0.05.

### PROP Thresholds and Efficacy of Blockers by Age Group and Genotype

**Figure 1:**
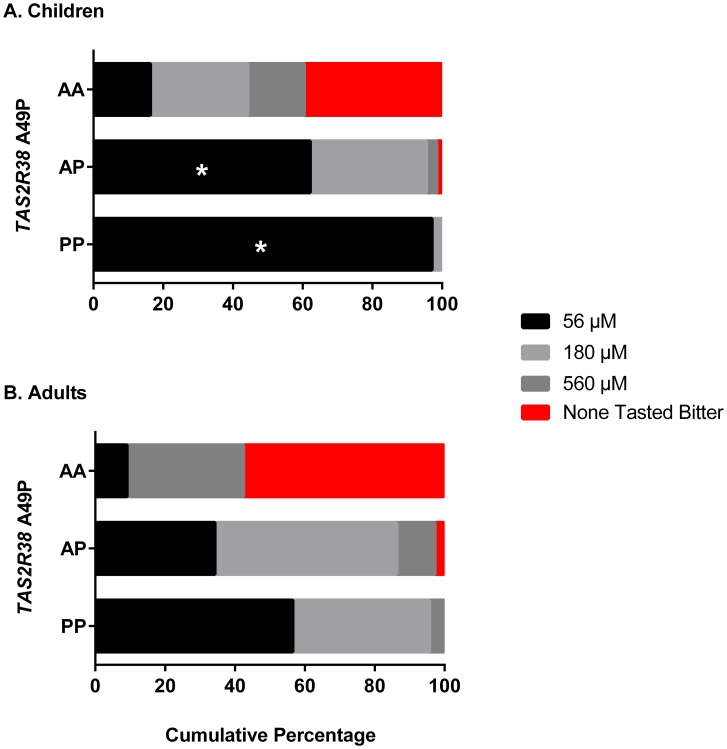
Effect of *TAS2R38* genotype on sensitivity to the bitter taste of PROP. The cumulative percentage of children (top) and mothers (bottom) in each of the three allele groups (AA, AP, PP) who first detected a bitter or “yucky” taste when sampling 56, 180 and 560 µM PROP or who never detected a bitter taste when sampling each of these PROP solutions (“none tasted bitter”). *Significantly different from adults of same *TAS2R38* genotype (p<0.05).

Genotypes at *TAS2R38* predicted PROP sensitivity, as determined by taste threshold measures, in both children and adults (**Figure 1**). The bitter-sensitive (P) form of the receptor gene displayed a heterozygote effect: two copies (PP) conferred greater PROP sensitivity than did a single copy (AP) in both children (p = 0.0004) and adults (p = 0.057). However, children who were heterozygous (AP) or homozygous dominant (PP) at *TAS2R38* A49P were significantly more likely to perceive the lowest concentration of PROP offered than were adults with the same genotypes (children vs. adults: AP: 62% vs. 34%; p = 0.001; PP: 97% vs. 57%; p = 0.005).

As shown in **Table 4**, the ineffectiveness of both blockers in reducing the bitterness of PROP was evident in each of the genotypes and for each age group. People homozygous for the bitter-sensitive P allele were no more and no less affected by the addition of a blocker than were heterozygotes. For people homozygous for the insensitive allele (A), PROP was not bitter and thus presented no opportunity for blocking.

## Discussion

Consistent with previous studies in adults [8], [12], [13], [14], [15], [16], [17], the ability of sodium salts to block bitter taste is compound specific and, as we show for the first time here, also specific to the age of the subject. In general, if the blocker worked for a given bitter in children, it also worked for adults, but not vice versa—in no case did a blocker work for children but not for adults. Further, if the bitterness for a particular compound was blocked by NaG in a given age group, then MSG was an effective blocker for that compound for that age group as well, but not vice versa. For only one bitter compound (caffeine) did NaG work but MSG did not. Overall, these results suggest that types of bitter receptors exist that respond differently based on the age of the subject and the chemical nature of the blocker and bitter agent.

The inborn rejection of bitter-tasting substances is thought to have evolved to protect the animal from being poisoned and the plant producing these chemicals from being eaten [2]. It may be that bitter receptors that detect poisons are most sensitive in childhood, when the risks of accidental poisoning are high [27]. To this point, denatonium (commonly called Bitrex) has a long history of being added to detergents and paints [28], [29] to deter pediatric poisonings, especially when used in conjunction with other preventive measures, such as child-resistant closures [30]. Unlike in adults, neither of the sodium salts under study was effective in blocking the bitterness of denatonium in children as a group. One explanation may be that children are more sensitive to its bitter taste than are adults.

To date, little research has assessed how children differ from adults in taste detection thresholds for bitter, with the exception of PROP [20]. Consistent with previous findings [20], [22], in our study children had lower detection thresholds than did adults, especially those with taste-sensitive genotypes. Therefore, blockers may be generally less effective for some children due to genotype because there is greater bitter taste to overcome. Unfortunately, we couldn't test this hypothesis for ratings of PROP solutions because neither blocker worked for that bitter compound. Our previous studies showed that the relationship between receptor genotype and bitter taste begins to resemble the adult pattern during mid-adolescence [22], and it may be that during childhood, a time of heightened bitter sensitivity to avoid accidental poisoning, this safety mechanism may fluctuate with age such that bitter sensitivity may peak at times when children begin making independent food choices. Further studies investigating both age- and genotype-related effects on blocker efficacy and bitter thresholds over time will be needed to pinpoint when and why some individuals are more bitter sensitive than others and how that changes with age. Such age-related effects extend beyond comparisons between children and adults since previous studies have shown that during adulthood there are significant compound-specific declines in bitter taste sensitivity [31], [32], [33] and detection of tastes in mixtures [33] between young and elderly subjects. Although foods and beverages having a prominent bitter taste component constitute a very small proportion of total consumption in industrialized countries [34], repeated exposure via dietary habits, learning and adaptation can shift hedonic responses to bitter-tasting foods and beverages and thus may also be key to understanding age-related effects [35].

Past research in adults suggests that when the two sodium salts NaG and MSG are effective in blocking bitter taste, they are acting at the level of peripheral taste receptors and not by central cognitive effects [12], [14], [16], [36], [37]. Because bitter compounds can stimulate multiple receptors [5], [38], the selectivity of NaG and MSG for specific bitter agents presumably arises because they stimulate different receptors that are more or less sensitive to their blocking effects. One suggestion is that for NaG or MSG, the allosteric effects of sodium ions might modify the activity of bitter receptors as it does for other G-protein-coupled receptors [39]. If the sodium is the active blocker, we would expect MSG to be less effective than NaG at the concentrations tested because it has less sodium. However, Keast and colleagues [15] found that the bitter-suppressing effect of MSG was not sodium dependent because increasing the MSG concentration from 10 to 100 mM did not reduce bitterness more. Thus, the mechanism whereby MSG blocks bitterness is not solely or perhaps even partially due to the sodium ion. Studies of non-sodium umami compounds are needed to understand its contribution *per se* to bitter blocking.

To our knowledge, this is the first study that has investigated whether PROP (the most widely studied bitter agent [40]), can be blocked by sodium salts. That it cannot be blocked by either NaG or MSG suggests that its receptor, *TAS2R38*, might be unaffected by sodium. However, we emphasize that when the blockers are effective, the exact nature their mechanisms of action remains unknown. Other supporting data for the peripheral effect of these bitter blockers comes from the ratings of liking for the blocker alone: we can rule out the possibility that the pleasantness of the blockers acted in the brain by distracting subjects from the negative aspects of bitterness, because children liked the taste of the NaG blocker more than adults, even though the blockers were less effective for them.

Studies that compare adults and children (especially the very young, like those studied here) require special measures to assure that the results are due to genuine sensory differences and not due to differences in children's ability to understand the task. The appropriateness of this method was confirmed in several ways. First, the majority of children understood and completed the task. Second, they were similar to adults in the ability of both blockers to reduce the bitterness of urea, thus suggesting that their responses were guided by the intensity of the bitter perception, not the complexity of the mixture (see also [8]). Third, as a further measure of validation, we took advantage of the relationship between the threshold measures of PROP and genotype of the *TAS2R38* receptor and confirmed not only the tight match between genotype and phenotype but also the effect of age on this relationship. Because the bitterness of PROP was not blocked by either blocker in either age group or genotype, we were prevented from examining bitter blocker efficacy by *TAS2R38* genotype. However, it is important to emphasize that, although paired-comparison methods can be used to investigate bitter blocking in children, there are limitations since this method cannot illustrate the magnitude of the efficacy of the blocker, as can other methods such as the general labeled magnitude scale [41].

In conclusion, drugs often taste very bitter, and this bad taste is a challenge to administering medications, especially liquid oral formulations for children [9]. Our results highlight the limitations of using adults to study the taste experiences of children [20], [42]. Adult taste panels do not represent the sensory worlds of children, and, as shown here, children do not receive the same benefit from bitter blockers as do adults. Consistent with a large body of research on age-related changes in sweet, salty, and bitter tastes [9], [22], [42], [43], the pattern of preferences was also different between the age groups: children preferred the taste NaG over water more than did the adults. Thus, children may like high-salt bitter blockers, as they do higher levels of sweetness [44], tastes that most adult taste panelists would reject. Furthermore, the methods used in the present study to assess the ability of blockers to reduce bitter taste represent a valuable tool for future assessment of taste in children, but we acknowledge that the need for more research on wider range of bitter stimuli including medicines as well as blockers in this area is acute [9], [45]. Future efforts should be directed at matching bitter medicines (ligands) to the appropriate bitter receptor(s) and the development of receptor-specific blockers

Children and adults are subject to many of the same ailments and diseases and, by necessity, are often treated with the same drugs. However, most of these drugs lack Food and Drug Administration approval for pediatric labeling for safety and efficacy [45]. Only a small fraction of these drugs, including their excipients, have been tested adequately for acceptability by pediatric populations, and many may not be appropriate for pediatric use [46], especially when many children cannot consume medication in solid oral dosage forms. The lack of “child-friendly” formulations leaves many of the world's children at increased risk for avoidable adverse events, including lack of adherence to medication regimens [1]. Any drug will be ineffective in children unless it is made in such a way that children will take it, and therefore a focus on taste and compliance is essential for pediatric medicine. More knowledge about the science of distaste and development of psychophysical methods that measure the human and especially the childhood taste experience will help realize a new era in drug development for children—an era that is still in its infancy [45]. Increasing knowledge about individual variation in taste due to both age and genetics will shed light on potential directions for developing personalized medicine (see also [47]).

## Supporting Information

Table S1 **Ranking procedure for paired-comparison data for one set of bitter stimulus-blocker pairings (urea-NaG): an example of hypothetical data from one subject.**
(DOCX)
