# Supplementary material for: Age-Related Differences in Bitter Taste and Efficacy of Bitter Blockers
Source: PLoS One. 2014 Jul 22;9(7):e103107. doi: 10.1371/journal.pone.0103107 (PMC4106902; doi:10.1371/journal.pone.0103107)
Supplement: Table S1 — Ranking procedure for paired-comparison data for one set of bitter stimulus-blocker pairings (urea-NaG): an example of hypothetical data from one subject. (DOCX) [file pone.0103107.s001.docx]

**Table S1.** Ranking procedure for paired-comparison data for one set of bitter stimulus-blocker pairings (urea-NaG): an example of hypothetical data from one subject.

**A. Paired comparison data (six possible pairings)**

| Stimulus chosen | vs. comparator stimulus |
| --- | --- |
| Urea | vs DI |
| Urea | vs Urea+NaG |
| Urea | vs NaG |
| NaG | vs DI |
| Urea+NaG | vs DI |
| Urea+NaG | vs NaG |

**B. Ranking procedure**

| Stimulus | Number of times chosen | Ranking |
| --- | --- | --- |
| Urea | 3 | 4 |
| Urea+NaG | 2 | 3 |
| NaG | 1 | 2 |
| DI | 0 | 1 |

Abbreviations: NaG, sodium gluconate; DI, deionized water. This table illustrates how data from a hypothetical subject for six possible pairings for a urea-NaG combination are ranked for statistical analysis, based on the number of times each stimulus was chosen. In this example, the bitter compound when offered alone is chosen most often when paired with other stimuli and is given the rank of 4. Friedman analyses of ranks were then conducted on ranking data for each bitter, each blocker, and each age group (children, adults) separately.
